# Practically Saline

**DOI:** 10.1177/2324709615618980

**Published:** 2015-11-27

**Authors:** Jonathan Schroeder, Catherine O’Neal, Tonya Jagneaux

**Affiliations:** 1 Louisiana State University Health Sciences Center, Baton Rouge, LA, USA

**Keywords:** *Empedobacter brevis*, septic shock, nonsterile intravenous fluid, contaminated saline

## Abstract

*Introduction*. In December 2014, the Food and Drug Administration issued a recall of all Wallcur simulation products due to reports of their use in clinical practice. We present a case of septic shock and multiorgan failure after the accidental intravenous infusion of a nonsterile Wallcur simulation product. *Case*. The patient presented with symptoms of rigors and dyspnea occurring immediately after infusion of Wallcur Practi-0.9% saline. Initial laboratory evidence was consistent with severe septic shock and multiorgan dysfunction. His initial lactic acid level was 9 mmol/L (reference range = 0.5-2.2), and he had evidence of acute kidney injury and markers of disseminated intravascular coagulation. All 4 blood culture bottles isolated multidrug-resistant *Empedobacter brevis*. The patient recovered from his illness and was discharged with ciprofloxacin therapy per susceptibilities. *Discussion*. This patient represents the first described case of severe septic shock associated with the infusion of a Wallcur simulation product. Intravenous inoculation of a nonsterile fluid is rare and exposes the patient to unusual environmental organisms, toxins, or unsafe fluid characteristics such as tonicity. During course of treatment, we identified the possible culprit to be a multidrug-resistant isolate of *Empedobacter brevis*. We also discuss the systemic failures that led to this outbreak.

## Introduction

**Figure 1.**
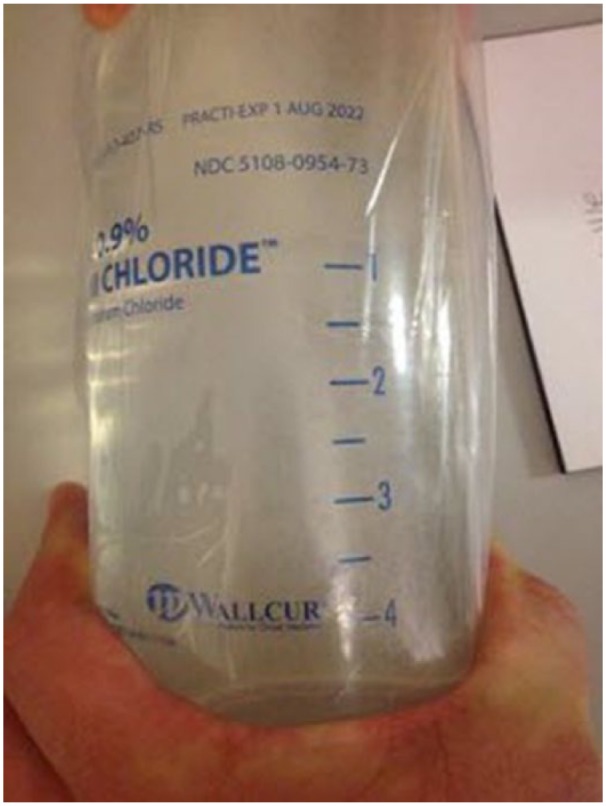
Image of recalled Practi-0.9% sodium chloride simulation fluid from the Centers for Disease Control report.

The administration of nonsterile intravenous fluids is rarely suspected or recognized in modern medical practice. However, in December 2014, the US Food and Drug Administration (FDA) reported 45 patients were exposed to Wallcur Practi-0.9% saline (Figure 1), a nonsterile fluid that is intended only for the simulation of intravenous infusion.^1^ Patients known to have received Wallcur products have experienced a febrile response during infusion and, in some cases, sepsis with end-organ dysfunction. There have been 25 adverse events, 11 hospitalizations, and 2 associated deaths.^1^ In this report, we present a clinical case of septic shock and multiorgan dysfunction associated with multidrug-resistant *Empedobacter brevis* bacteremia immediately following the infusion of Wallcur Practi-0.9% saline.

## Case Description

During an outpatient cosmetic procedure a 60-year-old male developed the sudden onset of confusion, rigors, tachypnea, and hypotension. The procedure, liposuction of the neck, lasted approximately 1 hour and was performed without generalized anesthesia. The only medications administered were local anesthesia with lidocaine, intravenous fluids, and 2 days of preoperative oral cephalexin. Immediately following the procedure, the patient developed rigors, chills, and nausea prompting concern of an adverse reaction or complication. The outpatient clinic transferred him to our emergency department via ambulance where he was found to be encephalopathic and hypotensive. His medical history indicated paroxysmal atrial fibrillation, cerebrovascular accident with mild dysarthria, and a bicuspid aortic valve. The patient’s medications included weekly testosterone injections, pregabalin, sildenafil, celecoxib, diazepam, esomeprazole, atorvastatin, dabigatran, and escitalopram. He had no reported drug allergies. Review of systems was negative for neck and chin pain, pruritus, or dyspnea. On examination, the patient was tachycardic, tachypneic, febrile, and diaphoretic. The chin and neck area exhibited a submental incision. Incisional edema, erythema, and tenderness were deemed appropriate for the immediate postprocedural time frame. Pulmonary auscultation revealed no abnormalities, and skin and mucosal exam revealed no rashes. Essential laboratory values (Table 1) were consistent with severe sepsis and multiorgan dysfunction. Initial electrocardiogram revealed atrial fibrillation with rapid ventricular response, which converted to sinus tachycardia following initial resuscitation. Computed tomography of the neck revealed small subcutaneous air collections without focal abscess. Computed tomography pulmonary angiography did not reveal evidence of pulmonary emboli. He was treated for presumed septic shock with 4 liters of normal saline. Central venous access was established and a norepinephrine infusion was initiated. Vancomycin and meropenem were administered empirically after blood and urine cultures were drawn. On transfer to the intensive care unit, his mentation improved and he was able to confirm that he had been asymptomatic prior to the procedure. Partial lactic acid clearance was documented after initial resuscitation (Table 1). Aspirin therapy was continued and stress dose corticosteroids were administered.

**Table 1.** Laboratory Values During the Course of Illness.

Laboratory Values	Hours Since Admission				
	0-6	6-12	24	48	Discharge
WBC (k/µL)	2.7	15	37	34	7.6
Neutrophils (%)	84	90	78	84	57
Lymphocytes (%)	12	2	2	5	27
Band forms (%)	2	7	15	10	6
Eosinophils (%)	0	0	—	—	10
Hct (%)	39	29	31	29	31
PLT (k/µL)	189	169	159	133	158
Erythrocyte count (M/µL)	5.32	—	—	—	4.8
Bicarbonate (mEq/L)	21	18	19	29	24
Urea nitrogen (mg/dL)	25	31	35	27	19
Creatinine (mg/dL)	1.8	2.1	2.2	1.3	0.9
Glucose (mg/dL)	123	96	—	—	—
Total bilirubin (mg/dL)	0.8	1.3	0.5	0.5	—
Alkaline phosphatase (U/L)	168	108	—	—	—
Aspartate aminotransferase (U/L)	36	50	84	75	—
Alanine aminotransferase (U/L)	54	76	127	115	—
Fibrinogen degradation products (µg/mL)	—	—	80 (<5)	—	—
Haptoglobin (mg/dL)	—	155 (40-240)	—	—	—
Lactate dehydrogenase (U/L)	—	367 (120-246)	—	—	—
Partial thromboplastin time (s)	34 (23-36)	41	41	37	—
Prothrombin time (s)	18 (12.3-14.6)	22.1	22.8	19	—
INR	1.5	1.9	2	1.6	—
Arterial blood gases					
pH	7.36	—	—	—	—
pCO_2_	26	—	—	—	—
pO_2_	77.3	—	—	—	—
O_2_Hb (%)	94				
Lactic acid (mmol/L)	9.9 (0.5-2.2)	4.5	6.2	—	—
Creatine kinase (U/L)	632	—	2963	—	—
Tr (ng/mL)	0.01 (<0.8)	1.79	2.59	—	—
Urinalysis	Bland	—	—	—	—
Urine drug screen	Negative	—	—	—	—
HIV-1/HIV-2	Negative	—	—	—	—
*Clostridium difficile* antigen	—	Negative	—	—	—

Abbreviations: WBC, white blood cell; Hct, hematocrit; PLT, platelet count; INR, international normalized ratio.

## Hospital Course

On hospital day 2, the patient reported subjective improvement in his symptoms despite increasing evidence of multiorgan dysfunction and disseminated intravascular coagulation. Adequate mean arterial pressures were still dependent on norepinephrine infusion despite a volume challenge, and his lactic acid remained elevated at 6.7 mmol/L. Serum creatinine increased to 2 mg/dL, and white blood cells increased to 37.6 k/µL. Antifungal therapy with micafungin was added empirically. A transthoracic echocardiogram revealed a depressed ejection fraction at 45%, no focal wall motion abnormalities, and a bicuspid aortic valve with no other significant valvular abnormalities. Our differential diagnoses narrowed to skin and soft tissue infection related to the procedure as well as exposure to contaminated intravenous fluids. The clinic physician had already contacted the FDA prompting a discussion with our intensive care unit team. The FDA confirmed an ongoing investigation of exposures to nonsterile intravenous fluids that were originally intended for training and simulation but had entered the supply chain for sterile fluids. On hospital day 3, the patient demonstrated continued hemodynamic and symptomatic improvement and was able to be transferred to a general medical floor. After 72 hours both sets of blood cultures grew isolates of *E brevis* (Table 2). Repeat blood cultures were negative. The patient was transitioned to oral ciprofloxacin and discharged with cardiology follow-up for monitoring of his bicuspid aortic valve.

**Table 2.** *Empedobacter brevis* Antibiotic Susceptibilities.

Antibiotic	MIC	Reported Resistance
Amikacin	>32	R
Aztreonam	>16	R
Cefepime	>16	R
Cefotaxime	>32	R
Ceftazidime	>16	R
Ceftriaxone	>32	R
Ciprofloxacin	≤1	S
Gentamycin	>8	R
Imipenem	≤4	S
Meropenem	>8	R
Piperacillin/tazobactam	>64	R
Tetracycline	≤4	S
Trimethoprim/sulfamethoxazole	≤2/38	S

Abbreviations: MIC, minimum inhibitory concentration; R, resistant; S, susceptible.

The outpatient surgical clinic confirmed they had recently purchased Wallcur Practi-0.9% saline from an online distributor under the assumption that it was a sterile fluid. Wallcur Practi-0.9% solution was used in their clinic at the time of our patient’s procedure and decompensation. The actual intravenous fluids administered in the clinic and during transport to the emergency department were discarded prior to our investigation. The clinic also confirmed they were currently complying with an FDA recall of Wallcur Practi-0.9% products.

## Discussion

The acuity of symptoms was unusual and narrowed our differential diagnosis. Hyperacute syndromes include severe septic shock, the Jarisch-Herxheimer reaction, type I hypersensitivity, acute intravascular hemolysis, and fat embolus syndrome. The presence of thrombocytopenia, anemia, coagulopathy, and elevated fibrin degradation products were suggestive of disseminated intravascular coagulation, a hallmark of sepsis. His symptoms and vital signs were most consistent with brisk activation of the innate immune system and cytokine storm. The growth of gram-negative organisms in 2 sets of blood cultures solidified the diagnosis of sepsis as the primary cause of his presentation.

We targeted our initial therapy toward maintaining adequate organ perfusion and treating for health care–associated infections. Antibiotics were selected to cover the resistant bacteria commonly experienced in health care settings such as methicillin-resistant *Staphylococcus aureus, Pseudomonas* species, and other resistant gram-negative bacteria including extended-spectrum β-lactamase-producing organisms. Persistent shock and his recent antibiotic usage prompted echinocandin therapy, although his overall risk for candidemia was low. Pathogenic molds are also a consideration in infusion-related infections; however, in this case antimicrobials targeting molds were withheld, as no mold forms were present on initial smears.

The organism cultured, *E brevis*, is an environmental, gram-negative, aerobic bacteria of the family Flavobacteriaceae. Reported pathogenicity has been rare and limited to health care exposures.^2,3^ The time to culture positivity was slow; however, the presence of an uncommonly cultured organism in both culture sets is suggestive of true bacteremia. The resistance mechanisms of *E brevis* have not been well-described,^4^ yet our subcultures displayed resistance to all classes of β-lactam antibiotics including the initial antibiotic regimen. Therefore, we suspect the patient’s recovery was largely due to maintenance of adequate organ perfusion and the low pathogenicity of *E brevis*.

Hyperacute presentations of sepsis are usually attributable to toxin-mediated diseases (ie, necrotizing fasciitis, toxic shock syndrome, the Jarisch-Herxheimer reaction, and meningococcemia); however, a sudden large exposure to bacterial cells and cell components would be expected to stimulate the pathogen recognition system responsible for the inflammatory phase of sepsis. Indeed, the experimental exposure of healthy subjects to endotoxin has shown to transiently induce the symptoms and hemodynamic effects of sepsis.^5^ This provides a partial explanation for this patient’s recovery in the setting of inadequate early antimicrobial coverage.

Accidental infusion of nonsterile fluids is extremely rare in developed countries and there have been no confirmed case reports in the United States since 1985.^6-8^ Wallcur Practi-0.9% saline is manufactured as a simulation product and thus the contents of the fluid are not regulated or advertised. Mechanisms of disease from exposure to a fluid of unknown composition include exposure to infectious agents (historically bacteria and molds), exposure to toxins (lipopolysaccharide/endotoxins, potassium, bacterial exotoxins), and exposure to nonisotonic solutions (hypotonic-free water). The FDA investigation into the contents of recovered Wallcur products revealed large amounts of endotoxin and high concentrations of environmental bacterial species.^9^ The investigators did not recover yeasts or molds.

## Conclusion

The infusion of a nonsterile intravenous fluid is rarely encountered in clinical practice. Even rarer is the infusion of a fluid that was not intended for human use. The medical literature provides no guidance on the treatment of these patients. Our patient had a life-threatening reaction to the infusion. We feel his positive outcome was primarily due to prompt resuscitation aimed at maintaining adequate perfusion. Empiric antibiotics were less likely to have affected his outcome as he had significant recovery before targeted therapy was achieved.

This is the first documented case of Wallcur Practi-0.9% Saline induced sepsis and *E brevis* bacteremia. The current outbreak of exposures to Wallcur Practi-0.9% Saline revealed a systematic failure in the manufacturing, distribution, and provider dispersion of intravenous saline and simulation products. As providers and distributors integrate new forms of distribution such as the Internet, this case raises important questions about the labeling, distribution, and regulation of intravenous fluids intended only for training and/or simulation, especially during the recent national shortage of normal saline. The FDA reports their investigation into their policies is ongoing.
